# Analysis of the potential dimensions and influencing factors of nurses’ sense of professional achievement in “Internet + Nursing Services”

**DOI:** 10.3389/fpubh.2025.1646405

**Published:** 2025-11-06

**Authors:** Jing Wu, Zhixia Zhang, Juanjuan Li, Heng Li, Niannian Zhang, Lili Gao, Congcong He, Xuan Wang

**Affiliations:** 1 Linfen Central Hospital, Linfen, China; 2 Changzhi Medical College, Changzhi, China

**Keywords:** “Internet + Nursing Services”, nursing, sense of career success, potential profile analysis, impact factor analysis

## Abstract

**Background:**

With the current development of smart technology and information in society, the integration of “Internet+” and medical services provides new solutions to tackle the current shortage of nursing resources. The feeling of professional success plays an important role in keeping the nursing workforce stable and improving the quality of nursing services. This study looks at "Internet + Nursing Services" and aims to explore the different types of professional success for nurses in this field. It also analyzes the key factors that shape each type in depth, providing a solid research foundation to improve the work environment for nurses and enhance the attractiveness.

**Methods:**

A convenience sampling method was used to select 378 nurses providing “Internet + Nursing Services” from six top-tier hospitals across different regions of Shanxi Province (two hospitals each from Jinbei, Jinzhong, and Jinnan) between August and October 2024. A questionnaire survey was conducted using general information forms, the Nurse Career Success Scale, the Psychological Capital Scale, the Work-Family Support Scale, the Perceived Social Support Scale, and the Nursing Work Environment Scale. We conducted a latent profile analysis to examine the career success of nurses providing “Internet + Nursing Services,” and variance analysis and multi-class logistic regression analysis were performed to analyze the factors influencing career success among these nurses.

**Results:**

The career success of the nurses providing “Internet + Nursing Services” was divided into three latent profiles: a low career success group (51 nurses, 13.5%), a medium career success group (201 nurses, 53.2%), and a high career success group (126 nurses, 33.3%). The analysis showed that marital status, per capita annual family income, work-family support, and psychological capital are the influencing factors of career success among the nurses providing “Internet + Nursing Services.”

**Conclusion:**

The career success of nurses providing “Internet + Nursing Services” showed significant differences across categories. We should develop targeted interventions based on the different characteristics and factors affecting each group to enhance the career success of these nurses.

## 1 Introduction

Currently, the aging population has become a significant global public health challenge. Countries such as the United States, the United Kingdom, Sweden, and Japan are seeing a steady rise in the demand for home care services and are vigorously promoting the development of electronic medical care and digital home care backed by information technology (1). China’s aging situation is also prominent: data from the seventh national census in 2020 showed that 18.7% of the total population is aged 60 and over, with those aged 65 and above making up 13.5% (2). This marks the official entry of China into an aging society. As the population of disabled, older, and empty-nest seniors continues to grow, there is a sharp increase in the demand for home care services among many older patients experiencing multiple chronic diseases.

Since the National Health Commission issued the “Internet + Nursing Services” pilot work plan, this new nursing model, supported by Internet information technology, has been vigorously promoted across the country (3). Among its various applications, home care services that rely on mobile Internet have shown significant value in the treatment of diseases in older patients, attracting widespread social attention. Relevant studies indicate that “Internet + Nursing Services” in China plays a key role in meeting the diverse needs of older patients with chronic diseases, improving their quality of life, and reducing readmission rates (4). However, the development of this model also faces real-world challenges, including insufficient nursing human resources, the need to boost nurses’ motivation, and issues such as the influence of negative emotions on order-taking behavior—all of which still require further exploration to identify solutions (5).

As key providers of home care services, nurses’ inner experiences and genuine feelings while participating in “Internet + Nursing Services” have been attracting increasing attention from scholars (6–9). A sense of professional success is a positive feeling that builds up over a person’s career, along with a sense of achievement gained through practice. As a core goal of professional development, it plays a crucial role in stabilizing the nursing workforce and improving the quality of nursing services (10). Therefore, nursing managers must focus on fostering nurses’ sense of professional success as a strategy to reduce turnover rates (11). Research shows that engaging in “Internet + Nursing Services” can enhance nurses’ sense of professional success and self-esteem; however, current related studies are mainly qualitative, with relatively few quantitative studies, and there is still a lack of consideration for population diversity.

Latent profile analysis is an individual-oriented approach that groups individuals with similar characteristics into the same profile, dividing them into different categories with significant characteristics to explore the traits and differences among different population categories, which aids in implementing targeted interventions (12). Applying this method to study nurses’ sense of professional success in “Internet + Nursing Services” allows for a deeper analysis of the population characteristics of these nurses and helps identify the factors influencing their professional success. Currently, there is a lack of latent feature analysis that specifically targets the professional success of nurses in “Internet + Nursing Services.” Therefore, there is significant potential for in-depth research using latent profiles.

In light of the above background, this study adopts latent profile analysis to systematically explore the sense of professional success among nurses in “Internet + Nursing Services.” The research aims to deeply analyze the different latent profile characteristics of nurses’ sense of professional success and accurately identify the key influencing factors that contribute to each profile. By constructing a scientific analytical framework and empirical research system, this study aims to provide a theoretical basis and data support for public health management departments. The goal is to formulate differentiated intervention strategies that enhance the sense of professional success of the nursing group involved in “Internet + Nursing Services.” This approach seeks to improve the talent development environment within the industry and promote the high-quality development of long-term care services.

## 2 Methods

### 2.1 Internet + Nursing Services

With the rapid development of electronic information technology, the Internet-empowered service system is becoming increasingly refined. The “Internet +” technology has deeply integrated into the healthcare sector. “Internet + Nursing Services,” which refers to the professional nursing services provided by registered nurses in medical institutions, uses online technology platforms and adopts an “online application and offline service” mechanism for discharged patients, those with special conditions, and individuals with limited mobility (13).

### 2.2 Design and participants

Using convenience sampling, a questionnaire survey was conducted among nurses providing “Internet + Nursing Services” in six tertiary Grade A hospitals (two each from Jinbei, Jinzhong, and Jinnan) in Shanxi Province from August to October 2024. The inclusion criteria were as follows: ① Registered nurses, ② Nurses who provided informed consent and participated voluntarily in this study, ③ Nurses involved in “Internet + Nursing Services” that are approved by the hospital, ④ Nurses working in Shanxi, and ⑤ Nurses providing at least one home nursing service. The exclusion criteria were as follows: ① Nurses who provided home care services without hospital approval, ② Nurses who previously engaged in home nursing services but are no longer working in the nursing profession, ③ Nurses who were on leave or otherwise unavailable, and ④ Nurses currently undergoing training. The sample size was determined to be 10–20 times the number of independent variables in the study. Since this study had a total of 20 independent variable items, the recommended sample size was between 200 and 400 cases. Considering a potential 10% invalid sample rate, the final sample size for this study was determined to be 220–440 cases. After removing the invalid questionnaires, we ended up with 378 cases. The hospital’s ethics committee approved this study (YP2024-40-1).

### 2.3 Measurements

#### 2.3.1 Sociodemographic characteristics

Based on the literature review, the research team designed this form, which included sex, age, marital status, department, years of work experience, monthly salary, average family income, number of night shifts, years of using Internet+, mode of transportation, and number of orders taken.

#### 2.3.2 Nurses’ Career Success Scale

Developed by Zhang et al. (14), this scale is used to assess the level of career success among nurses. The scale has a Cronbach’s *α* coefficient of 0.96. Its content validity index is 0.92, and the criterion-related validity is measured at 0.73. The scale includes 32 items across 4 key dimensions: intrinsic satisfaction (8 items), professional competence (9 items), external recognition (6 items), and career growth (9 items). Each item is rated on a scale from “strongly disagree” to “strongly agree,” with scores ranging from 1 to 5. This results in a total score ranging from 24 to 120. A higher score reflects a stronger sense of career success among nurses. In this study, the Cronbach’s *α* coefficient was 0.839.

#### 2.3.3 The Psychological Capital Scale

Developed by Luthans et al. (15) and translated by Chaoping Li, this scale is used to measure participants’ psychological capital. The Cronbach’s *α* coefficient of the scale is 0.923, and the ratio of the chi-square to degrees of freedom (x/df) is 3.92. The Comparative Fit Index (CFI) is 0.79, and the root mean square error of approximation (RMSEA) is less than 0.08. These results suggest that the model fits well overall. The scale consists of 24 items divided into 4 dimensions: hope (6 items), resilience (6 items), self-efficacy (6 items), and optimism (6 items). Each item is scored on a scale from 1 to 6, with responses ranging from “strongly disagree” to “strongly agree,” resulting in total scores between 20 and 120. The higher the score, the greater the psychological capital. In this study, the Cronbach’s *α* coefficient was found to be 0.846.

#### 2.3.4 The Work-Family Support Scale

Developed by Li et al. (16), this scale is used to measure the level of support individuals receive in the work and family domains. The Cronbach’s α coefficient for the scale is 0.82, with a ratio of x/df at 6.22. Additionally, the Goodness of Fit index (GFI), RFI, CFI, and NFI were all greater than 0.90, while the RMSEA is less than 0.08, which indicates that the model fits well overall. The scale has two parts: work support and family support. Work support is divided into organizational support (10 items) and leadership support (10 items), while family support includes emotional support (6 items) and practical support (items), making a total of 30 items across four dimensions. Each item is rated on a scale from “completely disagree” to “completely agree” with scores ranging from 1 to 5, resulting in total scores between 30 and 150. A higher score indicates that more support is received. In this study, we found the Cronbach’s *α* coefficient to be 0.827.

#### 2.3.5 The Perceived Social Support Scale

Developed by Zimet et al. (17) and translated and modified by Jiang (18), this scale is used to measure perceived social support levels among medical staff. The Cronbach’s α coefficient of the scale is 0.84, which shows that it has good reliability and validity. The scale consists of 12 items divided into 3 dimensions: family support (4 items), support from friends (4 items), and support from others (4 items). Each item is rated on a scale ranging from 1 to 7, where 1 means “strongly disagree” and 7 means “strongly agree,” with total scores ranging between 12 and 84. A score of 12–36 means low support, 37–60 means moderate support, and 61–84 means high support. In this study, we found the Cronbach’s *α* coefficient to be 0.883.

#### 2.3.6 The Nursing Work Environment Scale

Developed by Professor Lake at the University of Pennsylvania (19) and translated by Li and Lezhi (20), this scale is used to assess the characteristics of the nursing work environment. The Cronbach’s α coefficient for the scale is 0.910, and the content validity of the overall scale is 0.94. The scale includes 31 items across 5 dimensions: nurse participation in hospital affairs (9 items), the basis for high-quality service (10 items), competence and leadership style (5 items), adequate human and material resources (4 items), and medical-nursing collaboration (3 items). Each item is scored on a scale from 1 to 4, where 1 means “strongly disagree” and 4 means “strongly agree,” with total scores ranging between 31 and 124. A higher score indicates a better nursing environment. In this study, we found the Cronbach’s α coefficient to be 0.813.

### 2.4 Data collection and quality control

The researchers distributed the questionnaire using the Questionnaire Star platform. Before the survey, the researchers contacted the hospital administrators involved in the “Internet + Nursing Services” in Shanxi Province. The hospital administrators then shared the questionnaire link in the “Internet + Nursing Services” WeChat work group, explaining the inclusion and exclusion criteria to allow eligible participants to fill out the questionnaire. The questionnaire used standardized instructions to explain the research purpose, content, precautions, and confidentiality guidelines. After the questionnaires were collected, two researchers trained in the standardized process reviewed them, eliminating those that were completed in under 5 min or those with consistent or patterned answers.

### 2.5 Data analysis

This study used SPSS 26.0 for statistical description and analysis and Mplus 8.3 for latent profile analysis. First, the measurement data that were normally distributed were expressed as mean ± standard deviation, while categorical data were expressed as frequency and percentage. In the second step, we used Mplus 8.3 to conduct latent profile analysis of the nurses’ sense of career success in “Internet + Nursing Services,” starting with a single model and gradually increasing the number of latent profiles. We compared the fit indices of each model, including the Akaike information criterion (AIC), Bayesian information criterion (BIC), and adjusted Bayesian information criterion (aBIC); the smaller the index, the better the fit of the model. The entropy value indicates classification accuracy, with a value of ≥ 0.8 indicating greater precision as it approaches 1. The Lo–Mendell–Rubin adjusted likelihood ratio (LMR) and bootstrapped likelihood ratio test (BLRT) were used to assess differences in model fit, with a *p*-value of < 0.05 indicating that the model with k profiles outperformed the model with k-1 profiles. The smallest latent profile in the model should have a sample size of at least 5%. In the third step, univariate analysis of nurses’ sense of career success in “Internet + Nursing Services” was conducted using the chi-squared test, and multivariate analysis was performed using unordered multinomial logistic regression, with a p-value of < 0.05 considered statistically significant.

## 3 Results

### 3.1 General information of the survey participants and the Career Success Scale

A total of 400 questionnaires were collected for this study, with 378 valid responses, giving a response rate of 94.5%. The participants’ ages ranged from 22 to 60 years, with 98.4% being women and 1.6% men. A total of 82.8% of the nurses were married (*n* = 313), and more than half of the nurses had between one and three children (78.8%, *n* = 298). The majority of the nurses had at least a bachelor’s degree (97.9%, *n* = 370), and a high proportion of the nurses held intermediate or higher professional titles (69.7%, *n* = 263). The largest group of nurses had more than 10 years of work experience (60.5%, *n* = 229), and more than half of the nurses had an annual family income between 50,000 and 100,000 (55.6%, *n* = 210). Refer Table 1 for more details. The nurses scored an average of 88.69 ± 1.009 on the “Internet + Nursing Services” career success scale, with an average score of 30.44 ± 0.371 for the professional competence dimension, an average score of 18.14 ± 0.214 for the external recognition dimension, an average score of 17.35 ± 0.219 for the career growth dimension, and an average score of 22.75 ± 0.300 for the intrinsic satisfaction dimension.

**Table 1:** Univariate analysis of the potential profile classification of the nurses’ sense of professional success in “Internet + Nursing Service” (percentage,%).

Items (example)	Total number of cases (*n* = 378)	Low sense of professional success – low professional ability group (*n* = 51)	Medium sense of professional success – low career growth group (*n* = 201)	High career achievement – high intrinsic satisfaction group (*n* = 126)	Chi-squared value	*P*
Sex						
Male	6(1.6)	0(0)	2(1)	4(3.2)	3.306	0.191
Female	372 (98.4)	51 (100)	199 (99)	122 (96.8)		
Age						
≤25 years old	25 (6.6)	3 (5.9)	19 (9.5)	3 (2.4)	25.209	0.014
26–30 years old	68 (18)	7 (13.7)	46 (22.9)	15 (11.9)		
31–35 years old	98 (25.9)	11 (21.6)	55 (27.4)	32 (25.4)		
36–40 years old	123 (32.5)	18 (35.3)	51 (25.4)	54 (42.9)		
41–45 years old	56 (14.8)	11 (21.6)	27 (13.4)	18 (14.3)		
46–50 years old	4 (1.1)	0 (0)	1 (0.5)	3 (2.4)		
Over 51 years old	4 (1.1)	1 (2)	2 (1)	1 (0.8)		
Marital status						
Married	313 (82.8)	42 (82.4)	155 (77.1)	116 (92.1)	12.164	0.002
Unmarried	65 (17.2)	9 (17.6)	46 (22.9)	10 (7.9)		
Children number						
0	80 (21.2)	10 (19.6)	53 (26.4)	17 (13.5)	10.387	0.034
One	172 (45.5)	23 (45.1)	92 (45.8)	57 (45.2)		
Two or more	126 (33.3)	18 (35.3)	56 (27.9)	52 (41.3)		
Degree of education						
College degree or below	8 (2.1)	2 (3.9)	5 (2.5)	1 (0.8)	6.123	0.190
Undergraduate	362 (95.8)	46 (90.2)	193 (96)	123 (97.6)		
Master degree or above	8 (2.1)	3 (5.9)	3 (4.3)	2 (1.6)		
Professional title						
Primary nurse	115 (30.4)	12 (23.5)	71 (35.3)	32 (25.4)	11.138	0.084
Nurse-in-charge	16 (51.9)	24 (47.1)	102 (50.7)	70 (55.6)		
Deputy director of nurses and above	67 (17.8)	15 (29.4)	28 (13.9)	24 (19)		
Monthly income						
≤3,000	38 (10.1)	2 (3.9)	26 (12.9)	10 (7.9)	6.746	0.150
3,000–6,000	375 (72.8)	36 (70.6)	144 (71.6)	95 (75.4)		
≥6,000	65 (17.2)	13 (25.5)	31 (15.4)	21 (16.7)		
Working years						
≤5 years	68 (18)	9 (17.6)	44 (21.9)	15 (11.9)	17.388	0.026
6-10 years	81 (21.4)	8 (15.7)	53 (26.4)	20 (15.9)		
11-15 years	137 (36.2)	17 (33.3)	63 (31.3)	57 (45.2)		
16-20 years	61 (16.1)	12 (23.5)	28 (13.9)	21 (16.7)		
≥21 years	31 (8.2)	5 (9.8)	13 (6.5)	13 (10.3)		
Average annual income per family						
< 50,000	112 (29.6)	10 (19.6)	78 (38.8)	24 (19)	4.830	0.008
50,000 to 100,000	210 (55.6)	33 (64.7)	104 (51.7)	73 (57.9)		
> 100,000	56 (14.8)	8 (15.7)	19 (9.5)	29 (23)		
Number of night shifts per month						
≤1 time	116 (30.7)	20 (39.2)	50 (24.9)	46 (36.5)	14.488	0.025
2–5 times	65 (17.2)	12 (23.5)	31 (15.4)	22 (17.5)		
5–8 times	147 (38.9)	14 (27.5)	85 (42.3)	48 (38.1)		
≥8 times	50 (13.2)	5 (9.8)	35 (17.4)	10 (7.9)		
Mode of transportation for medical appointments						
Specialized transportation to and from the hospital platform	37 (9.8)	6 (11.8)	17 (8.5)	14 (11.1)	13.391	0.099
Self-driving	179 (47.4)	27 (52.9)	82 (40.8)	70 (55.6)		
Take a taxi	89 (23.5)	13 (25.5)	53 (26.4)	23 (18.3)		
Transportation by patients’ families	3 (0.8)	0 (0)	2 (1.6)	1 (0.8)		
Other	70 (18.5)	5 (9.8)	47 (23.4)	3 (0.8)		
Number of orders per month						
≤1time	287 (75.9)	41 (80.4)	162 (80.6)	84 (66.7)	13.139	0.041
2–4 times	83 (22)	8 (15.7)	35 (17.4)	40 (31.7)		
5–8 times	6 (1.6)	2 (3.9)	3 (1.5)	1 (0.8)		
More than 8 times	2 (0.5)	0 (0)	1 (0.5)	1 (0.8)		
Services performed after becoming an online nurse						
Primary nursing service	256 (67.7)	38 (74.5)	140 (69.7)	78 (61.9)	3.368	0.186
Specialized nursing service	122 (32.3)	13 (25.5)	61 (30.3)	48 (38.1)		

### 3.2 Analysis of the potential profiles of nurses’ career success in “Internet + Nursing Services”

This section presents an analysis of the potential profiles related to nurses’ career success in the context of Internet + Nursing Services. We sequentially established between 1 and 4 potential profile models to analyze the career success of the nurses in “Internet + Nursing Services.” As the AIC, BIC, and aBIC values increased, they gradually decreased. When the model number was 4, the LMR(p) value was > 0.05; when the model number was 3, the AIC, BIC, and aBIC values tended to stabilize, where the LMR(p) and BLRT(p) were both less than 0.05, entropy was > 0.8, and each profile probability was greater than 5%. A comprehensive comparison of the fit indices of each model indicated that the 3-model was the best-fitting model. See Table 2.

**Table 2:** Fitting indexes of the potential profile models for nurses’ professional success in “Internet + Nursing Services.”

Model	AIC	BIC	aBIC	LMR(p)	BLRT(p)	Entropy	Category probability(%)
1	4013.163	4044.642	4019.260	-	-	-	1.000
2	3178.108	3229.262	3188.016	<0.001	<0.001	0.919	0.198/0.802
3	2750.227	2821.055	2763.945	<0.001	<0.001	0.886	0.135/0.532/0.333
4	2609.112	2699.614	2626.640	0.157	<0.001	0.889	0.127/0.175/0.437/0.262

### 3.3 Analysis of the characteristics of the potential categories of nurses’ sense of professional accomplishment in “Internet + Nursing Services”

**Figure 1:**
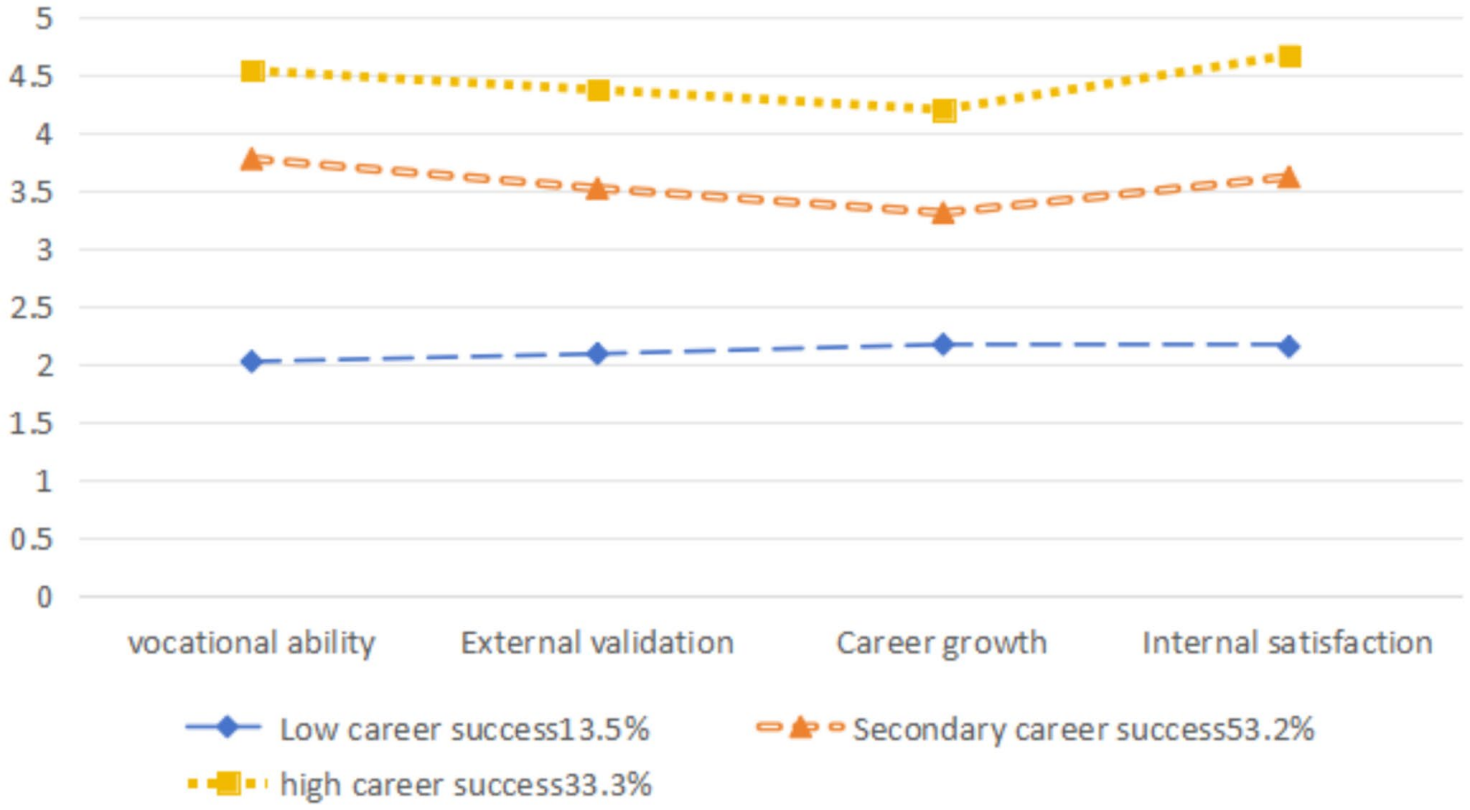
Distribution of features of three potential profiles of sense of professional success among the nurses in “Internet + Nursing Services”.

According to the results of the latent profile analysis, the scores of “Internet + Nursing Services” across various dimensions are illustrated in Figure 1. Category 1 scored low across all dimensions, with the lowest score in the professional skills dimension, referred to as the low professional success group. This group consisted of 51 people, accounting for 13.5% of the study participants. Category 2 scored at a moderate level across all dimensions, with the lowest score in the career growth dimension, referred to as the medium professional success group. This group consisted of 201 people, accounting for 53.2% of the study participants. Category 3 scored high across all dimensions, with the highest score in the intrinsic satisfaction dimension, referred to as the high professional success group. This group consisted of 126 people, accounting for 33.3% of the study participants.

### 3.4 Single-factor analysis of possible types of the nurses’ sense of professional success in nursing services during the internet era

There were significant differences among the three potential profiles of the nurses in “Internet + Nursing Services.” These differences included age, marital status, number of children, years of work experience, average household income, average monthly night shifts, and number of cases handled, as shown in Table 1.

### 3.5 Multifactor analysis of the potential categories of career success among the nurses in “Internet + Nursing Services”

This study explores the factors affecting nurses’ career success in the context of Internet-based nursing services. Using the three potential profiles of “Internet + Nursing Services” as dependent variables, the variables that showed statistically significant differences in the univariate analysis were considered independent variables, with the values assigned to the independent variables listed in Table 3. The scores for the Psychological Capital Scale, Work-Family Support Scale, Perceived Social Support Scale, and Nursing Work Environment Scale were replaced with their actual values. The results of the logistic regression analysis indicated that marital status, per capita annual income of the family, work-family support, and psychological capital influence nurses’ career success in “Internet + Nursing Services” (*p* < 0.05), as you can see in Table 4.

**Table 3:** Variable assignment table.

Independent variable	Assignment method
Nurses’ sense of professional success	Low level of professional success = 1；middle-level sense of professional success = 2； high level of professional success = 3；
Age	≤25 years old = 1; 26–30 years old = 2; 31-35 years old = 3; 36-40 years old = 4; 41-45 years old = 5; 46-50 years old = 6; ≥ 51 years old = 7
Marriage	married = 1; unmarried = 2
Number of children	0个 = 1; 1个 = 2; ≥ 2个 = 3;
Working life	<3 years = 1; 3-5 years = 2; 6-8 years = 3; >8 years = 4
Average annual income per family	<50,000 = 1; 50,000 to 100,000 = 2; > 100,000 = 3
Number of night shifts per month	≤1time = 1; 2-4times = 2; 5-7times = 3; ≥ 8times = 4;
Years of Internet + Nursing Services	<3 years = 1; 3-5 years = 2; 6-8 years = 3; >8 years = 4
Number of orders received per month	≤1time = 1; 2-4times = 2; 5-8times = 3; >8times = 4
Work-family support	Measured value
Nursing working environment	Measured value
Psychology capital	Measured value
Perceived social support	Measured value

**Table 4:** Multivariate analysis of the potential profiles of the nurses’ sense of professional success in “Internet + Nursing Services.”

Item	Low sense of professional success – low professional ability			High career achievement – high intrinsic satisfaction		
	OR	OR95%	*P*	OR	OR95%	*P*
Marital status^a^						
Married	1.458	0.228–9.325	0.039	0.151	0.027–0.837	0.031
Average annual family income^b^						
<50,000	0.438	0.124–1.551	0.000	3.372	1.171–9.710	0.024
>100,000	0.857	0.303–2.424	0.042	2.400	0.984–5.851	0.044
Work-family support score	1.043	1.015–1.072	0.003	1.262	1.036–1.389	0.000
Psychology capital score	0.944	0.963–1.026	0.000	1.047	1.018–1.176	0.001

Referencing the middle-level career success group as a benchmark. Annotation a refers to “unmarried” as the reference group, and annotation b takes “50,000–100,000 RMB as the reference.

## 4 Discussion

This study found that the sense of professional success among nurses in “Internet + Nursing Services” can be divided into three potential profiles: low success group, medium success group, and high success group, indicating significant heterogeneity in their sense of professional success. The analysis is as follows: (1) The low success group had a relatively low overall sense of professional success, accounting for 13.5%. Compared to working in hospitals, providing home care services actually places greater demands on nurses’ specialized skills due to the change in work setting and environment, the complexity and variability of patients’ conditions, the lack of support from medical teams, and the lack of timely access to emergency medical supplies and equipment. We need to enforce strict qualification and access requirements, establish a unified training and assessment system (21), review the challenges faced during home services and our responses, summarize and share classic cases, and continuously learn to improve home nursing service capabilities. (2) The medium success group accounted for 53.2%, showing that the nurses in “Internet + Nursing Services” generally felt moderately successful. This group had the lowest scores in the professional growth dimension. Research shows (22) that leadership is an important factor in professional growth; leaders can improve employees’ mental models and tap into their potential through motivation and inspiration, fostering mutual development for both individuals and organizations through self-transcendence. Nursing managers should focus on helping nurses grow by providing guidance and more practice opportunities to promote their professional development. (3) The high success group accounted for 33.3%, and this group scored the highest in the dimension of intrinsic satisfaction. The sense of organizational support is an important predictor of the fulfillment of individuals’ basic psychological needs (23). Hospital managers should provide sufficient organizational support for nurses in home care services, fully consider nurses’ strengths, and utilize these strengths to enhance their sense of intrinsic satisfaction. Nurses themselves should also continuously pursue self-realization and alignment of values, as well as personal growth, to enrich their personal lives.

Our research also aimed to explore the factors influencing the sense of professional success among nurses working in “Internet + Nursing Services.” This study found that marital status, average family income, work-family support, and psychological capital are the main factors influencing this group’s professional success. The results indicate that unmarried nurses are more likely to fall into the low professional success group. Professional skills refer to the abilities and techniques required to complete a task, and they also involve the relevant environment and individual motivation (24). Rotter categorizes professional skills into self-competence, professional competence, methodological competence, and social competence (25). Unmarried nurses may be younger, and their self-awareness, planning, and management skills might not have fully developed yet. They tend to have less work experience and weaker professional and problem-solving skills, along with a lack of social experience and communication abilities. In addition, the home care environment is more complicated, and unmarried nurses might experience less financial pressure, leading to lower motivation to participate in “Internet + Nursing Services.” Furthermore, unmarried nurses may be under pressure from societal role expectations, face challenges in maintaining work-life balance, and lack a complete emotional and interpersonal support network, all of which impact how successful they feel in their careers. Therefore, managers should not only focus on enhancing nurses’ professional competence but also guide the career planning of young unmarried nurses, foster problem-solving skills, and improve communication abilities, while being more attentive to their personal lives and offering more emotional support (35).

The results of the logistic regression analysis in this study indicate that nurses with an annual per capita income of over 100,000 yuan are more likely to belong to the high career success group. Income level is a key indicator of personal career success and reflects the value that hospitals and departments assign to nurses’ professional abilities, which significantly influences nurses’ sense of career success (27). Related research shows that the more satisfied nurses are with their income, the more they recognize their own value, which, in turn, enhances their sense of career success (28). Therefore, it suggests that hospitals and nursing managers should further refine their performance-based salary assessment systems, effectively increase nurses’ benefits and salaries, and boost their enthusiasm for working in “Internet + Nursing Services” by reasonably increasing nurses’ income. At the same time, they should expand the “Internet + Nursing Services” projects, boost the revenue from “Internet + Nursing Services,” and enhance individual nurses’ sense of career success (29).

The results of this study indicate that the higher the work-family support score, the greater the likelihood of being classified in the high career success group, which aligns with the findings of He et al. (26). Work-family support refers to the support individuals receive in balancing, managing conflicts between, and expanding work and family relationships, and it is a significant predictor of psychological health (30, 31). Nurses participating in Internet + Nursing Services mainly utilize their break time, with time and physical risk perceptions being the main risks associated with Internet + Nursing Services (32). Family support can promote individuals’ recovery, help maintain physical and mental health, and enhance overall well-being (33). Work support can strengthen nurses’ sense of belonging, stimulate their work potential, encourage proactivity and initiative, and increase work engagement (36). At the same time, work-family support is also an intrinsic motivator for nurses; when facing significant work pressure, nurses can receive understanding and encouragement from the organization, leadership, and family, offering positive guidance (34). Therefore, it is essential to strengthen the promotion of “Internet + Nursing Services,” providing positive guidance to help the public recognize the role and value of nurses in this work. As part of the larger community, nurses’ families can provide greater support and understanding for their involvement in “Internet + Nursing Services,” alleviating their concerns and enhancing their sense of career success. Hospital managers should provide sufficient support for nurses’ participation in “Internet + Nursing Services,” implement flexible scheduling, reasonably arrange work time and tasks, and set up a solid support system for technical assistance when facing challenges during home services (34, 37).

This study found that psychological capital is a potential influencing factor in nurses’ sense of career success, with the nurses in the “low career success” group scoring the lowest in psychological capital, while those in the “high career success” group scored the highest. This psychological resource is an individual’s intrinsic positive psychological asset that can energize them in their work, improve their work behavior, and further influence their work attitude and professional abilities (38). Ma (39) research on 248 clinical nurses found a positive correlation between nurses’ psychological capital and their sense of career success. Chang et al. (40) implemented a personal and group psychological assistance program for 290 nurses, using psychological knowledge for a unified intervention, and the findings revealed a significant increase in nurses’ career success scores after the intervention. This might be linked to aspects of psychological capital, such as confidence, hope, and resilience. These positive psychological states can influence individuals to strive toward higher career goals, thereby enhancing nurses’ sense of career success. Furthermore, the higher the sense of career success, the greater the nurses’ ability to adapt professionally and handle stress (41), ensuring the sustainability of Internet-based nursing services. Therefore, nursing managers should focus on developing nurses’ psychological capital, promote the value transformation of this psychological resource, regularly conduct surveys and analyses of nurses’ psychological capital, and implement relevant measures, such as psychological workshops (42), group support, and stress management training, to further enhance nurses’ sense of career success.

## 5 Limitations

Although this study provides a preliminary exploration of the sense of professional success among nurses in “Internet + Nursing Services,” it still has certain limitations related to factors such as research design, sample characteristics, and variable selection. First, this study used a cross-sectional survey, which can only present a snapshot at a specific point in time and cannot track how the sense of professional success changes over time, and it also cannot clarify how the variables are related. Furthermore, the sample size was pretty small, which might have impacted the representativeness of the results. Future research could conduct large-sample longitudinal studies. Second, this study did not break down the service content, only conducting a general discussion; subsequent research could perform a detailed analysis based on the specific content of “Internet + Nursing Services” to make the study more accurate. Third, this study only examined the direct effects of related factors on the results, overlooking how different factors might indirectly affect things. Future research could use structural equation modeling to explore, in depth, the factors influencing professional success among nurses in “Internet + Nursing Services.”

## 6 Conclusion

This study, through the latent profile analysis method, divides the sense of career success among nurses in “Internet + Nursing Services” into three groups: low, medium, and high career success. The results indicate that marital status, per capita family income, psychological capital, and work-family support are the main factors affecting their career success. This research enhances our understanding of career success among nurses in “Internet + Nursing Services” and suggests that we should pay more attention to new nurses and those who feel less successful in their careers. By providing targeted support and guidance, more nurses can be encouraged to get involved in the field of Internet + Nursing Services, helping the “Internet + Nursing Services” model grow sustainably and ensuring that the growing demand for convenient nursing care is met as our society ages.

## Data Availability

The raw data supporting the conclusions of this article will be made available by the authors, without undue reservation.
